# Regional Lymph Node Metastasis Distribution in Resectable Middle-Third Gastric Cancer: A Cross-Sectional Study

**DOI:** 10.7759/cureus.41236

**Published:** 2023-06-30

**Authors:** Nguyen Vu Tuan Anh, Quang Thong Dang, Nguyen Lam Vuong, Viet Hai Nguyen, Le Minh Quoc Ho, Quang Dat Tran, Truong Thai Dang, Anh Minh Tran, Thuy Nguyen Doan, Hoang Bac Nguyen, Trung Tin Nguyen, Long Duy Vo

**Affiliations:** 1 General Surgery, University of Medicine and Pharmacy at Ho Chi Minh City, Ho Chi Minh City, VNM; 2 Gastrointestinal Surgery, University Medical Center at Ho Chi Minh City, Ho Chi Minh City, VNM; 3 Medical Statistics and Informatics, Faculty of Public Health, University of Medicine and Pharmacy at Ho Chi Minh City, Ho Chi Minh City, VNM

**Keywords:** gastric lymph node distribution, near-total gastrectomy, sub-total gastrectomy, middle-third gastric cancer, lymph node mapping

## Abstract

Introduction

Lymph node (LN) metastasis happens even in early gastric cancer (GC) even in LN stations that are not adjacent to the primary tumor. Total or subtotal gastrectomy (TG or sTG) can be performed in the middle third of the GC if the negative proximal margin is maintained. These procedures differed in the extent of LN dissection; therefore, oncology considerations must be taken into consideration when selecting the appropriate procedure.

Methods

This was a cross-sectional study involving 98 patients suffering from middle-third GC. The metastatic lymph nodes (mLN) ratio was calculated in each case by the ratio between the number of mLN and the number of total LNs retrieved. We compare the difference in the total LN retrieved, number of mLN, and rate of positive LN (N+) between the two groups TG and sTG.

Results

The majority of patients had advanced GC (82.7% pT2-4). About 65.3% of patients had metastasis LN. The events of LN metastasis and skipped LN metastasis happened even in tumors contained in the submucosal layer. The metastasis rates in each LN station were also increasing in correlation with the depth of tumor invasion. For LN station No. 2, 4sa, 10, 11d (which are not mandatory) in sTG, the rate of mLN was 0% for the pT1-3 tumor, regardless of tumor longitudinal location. The rate of mLN for each station was higher in adjacent stations of the tumor (No. 1-3-5-7 in lesser curvature, No. 4sb-4d-6 in greater curvature, No.1-3-4sb in the anterior wall, No. 3-7-12a in the posterior wall). The total LN retrieved, number of mLN, and rate of positive LN were statistically higher in the TG group compared to the sTG group. However, the mean mLN ratios between the two groups were comparable (p = 0.116).

Conclusion

In accordance with the macroscopic and microscopic characteristics, we observed a stratified distribution of mLN in the middle third of the GC. With these early results, sTG combined with standard lymphadenectomy was an acceptable treatment for T1-T3 middle-third GC in terms of mLN distribution. Total No. 4sb LN dissection might also be reserved in gastrectomy for T1-T3 GC.

## Introduction

Gastric cancer (GC) is one of the most prevalent malignancies and digestive tract diseases. In terms of annual incidence, GC was ranked as the fourth most common cancer and the second most frequent among gastrointestinal cancers [1]. Even in early GC that does not invade the mucosal or submucosal layer (T1) and in LN stations that are not adjacent to the tumor, LN metastasis occurs in GC. The rate of LN metastasis in early GC was 9.7%-12.3% in the previous study [2]. In advanced GC with a further invasion of the submucosal layers, the LN metastasis rate can be extremely high (up to 50% for T2-3 tumors) and rises to 80%-90% in late-stage GC [3]. The total number of retrieved LN and the LN metastasis rate is proven to be two of the most crucial factors that affect treatment planning and long-term prognosis [3,4].

Recent guidelines for the management of GC recommend that the treatment plan is based on a multidisciplinary approach, with gastrectomy and sufficient LN dissection being the most important parts. The type of gastrectomy depends on multiple factors, in which the location of the tumor is the main aspect that determines the range of gastrectomy and LN dissection. In the middle-third GC, which was included in 14.4%-38.0% of GC in the previous studies [5], total gastrectomy (TG) or subtotal gastrectomy (sTG) can be conducted if the negative proximal margin is guaranteed. These procedures varied in their range of LN dissection, so the determination of the applied procedure has to be careful in considering oncology aspects [6].

Based on data from the distribution of metastatic lymph nodes (mLN) after macroscopic and microscopic findings of GC, the LN stations needed for LN dissection for each type of gastrectomy have been added to Japanese guidelines for the treatment of GC [4,6]. However, these guidelines have been recently revised and updated on a regular basis. Thus, we aimed to evaluate the distribution of LN metastasis based on macroscopic and microscopic findings of GC in the middle third of the stomach. Our primary outcomes were the mLN rates at each station according to the depth of invasion and the location of the tumor in order to improve the indication for sTG, which is also in the trend of function-preserving gastrectomy for GC.

## Materials and methods

Study design and population

This was a single-center cross-sectional study from January 2017 to December 2021 at University Medical Center at Ho Chi Minh City, a tertiary referral hospital in Vietnam. Patients were selected from our surgery registry system following these criteria: (i) patients who underwent curative laparoscopic gastrectomy with standard lymphadenectomy according to Japanese guidelines for the management of gastric cancer, version 5, 2018 [4] and (ii) LN metastasis was evaluated for each station according to our process (mentioned below). The exclusion criteria were (i) concurrent cancer or history of previous cancer and (ii) neoadjuvant chemotherapy. A multidisciplinary consultation determined the patient's final course of treatment, including whether surgery was an option. Based on those criteria, 98 patients were chosen for this study. Written informed consent was obtained from the patient for publication of this cross-sectional study and accompanying images.

Surgical technique and histological examination of resected specimens

Both sTG and TG complied with the principles of the extent of distal gastrectomy and D2 LN dissection according to the Japanese guidelines [4].

First, the total greater omentum was removed from the transverse colon. The dissections continued to the root of the left gastro-epiploic artery. The connective tissue was removed until the avascular region between the gastro-epiploic artery and short gastric arteries (group #4sb). Then, we dissected the connective tissue from the inferior border of the pancreas and the root of the right gastro-epiploic artery. The infra-pyloric artery was then exposed and ligated. The connective tissue from the right side of the right gastro-epiploic artery to the greater curvature side of the duodenum was removed (LN groups #6a, #6v, and #6i). Next, the adipose tissues along the celiac trunk, common hepatic artery, and proximal part of the splenic artery (including LN groups #9, #8a, and #11p) were dissected. The dissection was continued to the left side of the proper hepatic artery. The right gastric artery (including LN group #5) was exposed and ligated. The tissue including LN group #12a was dissected down to the left side of the portal vein. The dissection was continued to the avascular region on both sides of the left gastric vessels to expose the root of the left gastric artery from the celiac trunk and the left gastric vein. Then, the left gastric artery (including LN group #7) was ligated. The connective tissue in front of the right diaphragmatic crus and tissue along the lesser curvature was removed (LN groups #1 and #3).

**Figure 1:**
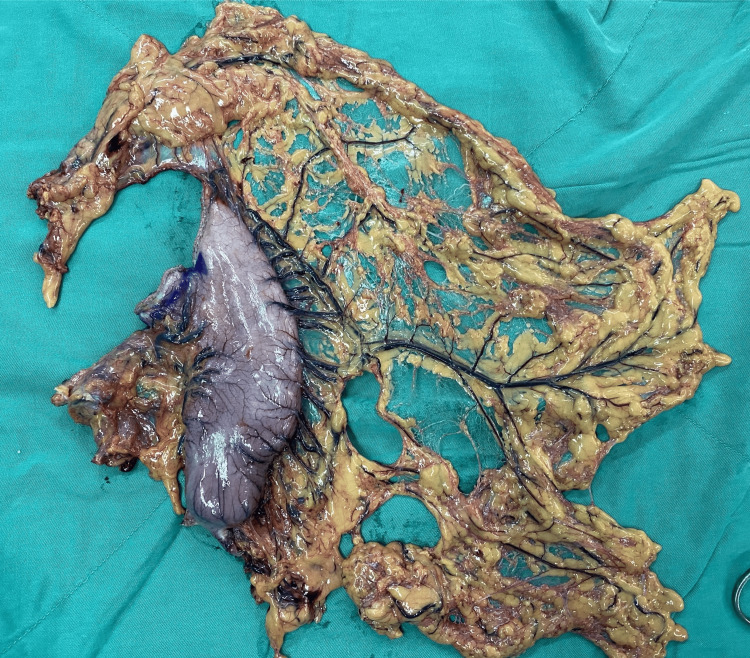
The en-bloc postoperative specimen

Finally, the stomach was transected using a linear stapler (blue cartridge) above the tumor edge 3-5 cm (3 cm for type 1-2, and 5 cm for type 3-4). Intraoperative esophagogastroscopy would be performed to confirm the upper tumor edge and proximal margin, in cases of tumor sT1-2 or tumor type 3-4. In cases of ensuring an R0 proximal margin to preserve a small gastric remnant, we performed sTG. Otherwise, TG was chosen, and LN dissection was continued along the splenic artery up to the splenic hilum to remove groups 11p and 10. Short gastric arteries would then be ligated; connective tissues around them and the left side of the cardia were removed (including LN group #2, 4sa). The en bloc specimen would be placed in a bag and removed from the abdomen (Figure 1).

**Figure 2:**
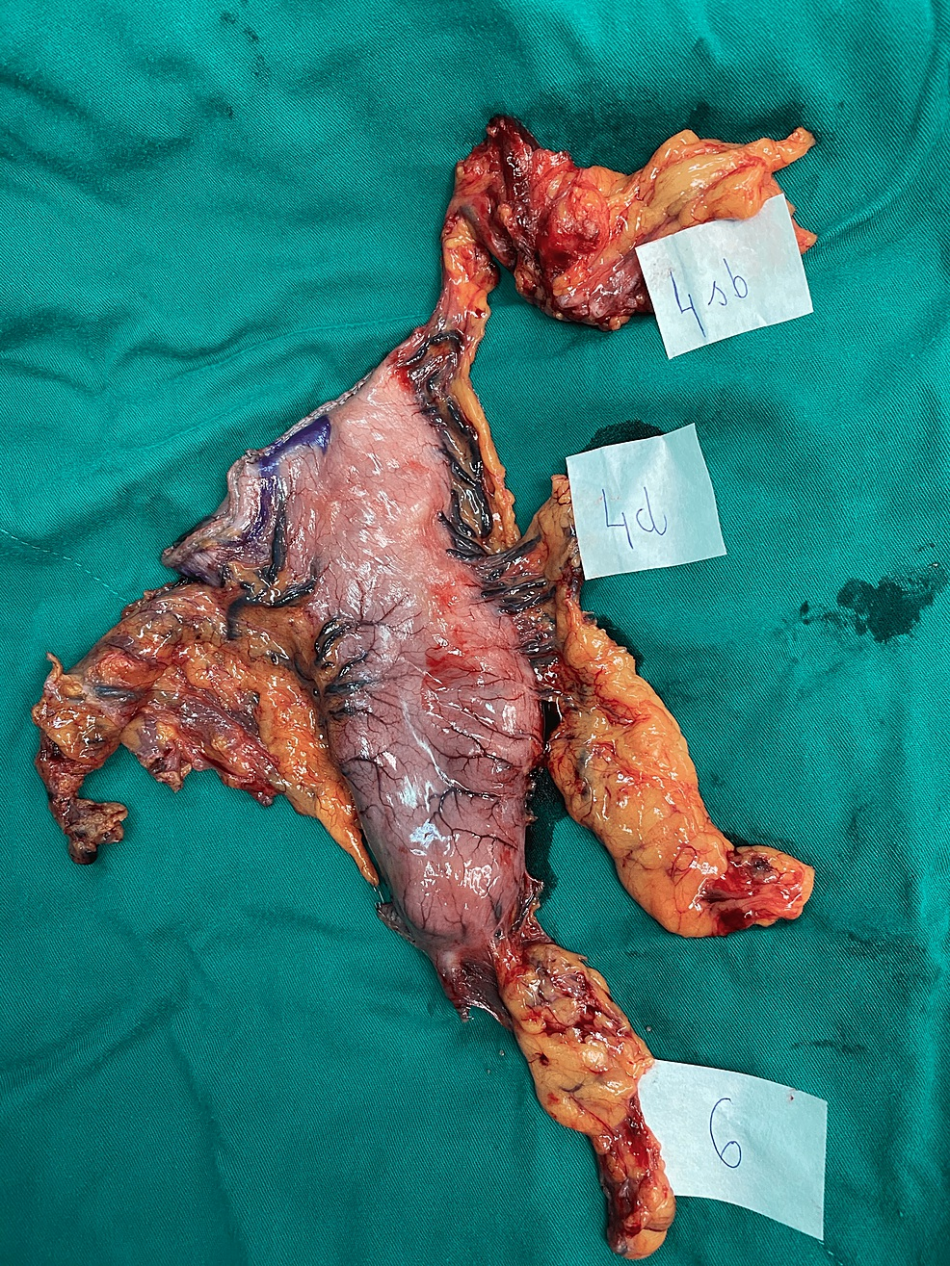
Lymph node station No. 4sb, No. 4d, and No. 6 from the specimen

**Figure 3:**
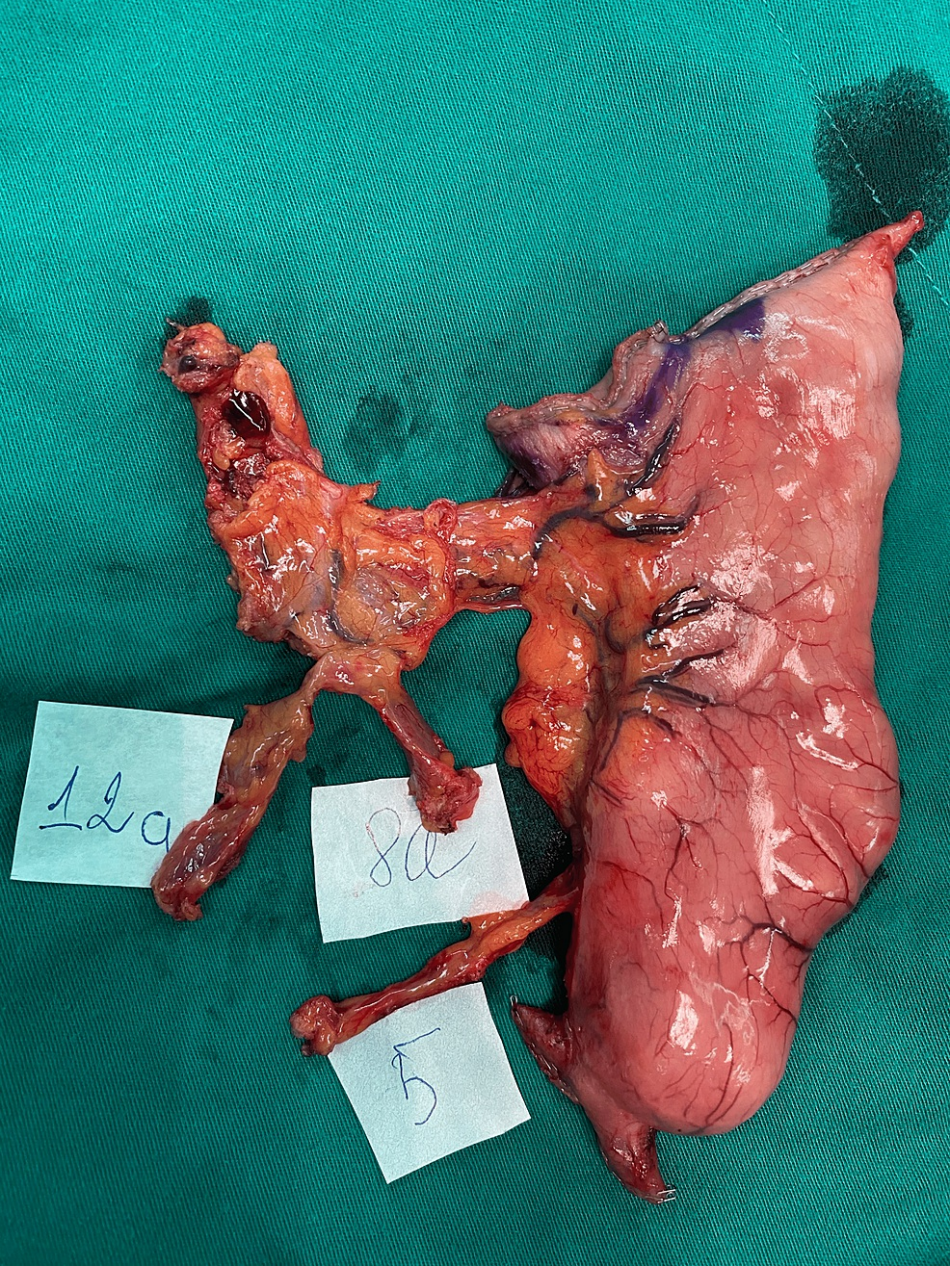
Lymph node station No. 5, No. 8a, and No. 12a from the specimen

**Figure 4:**
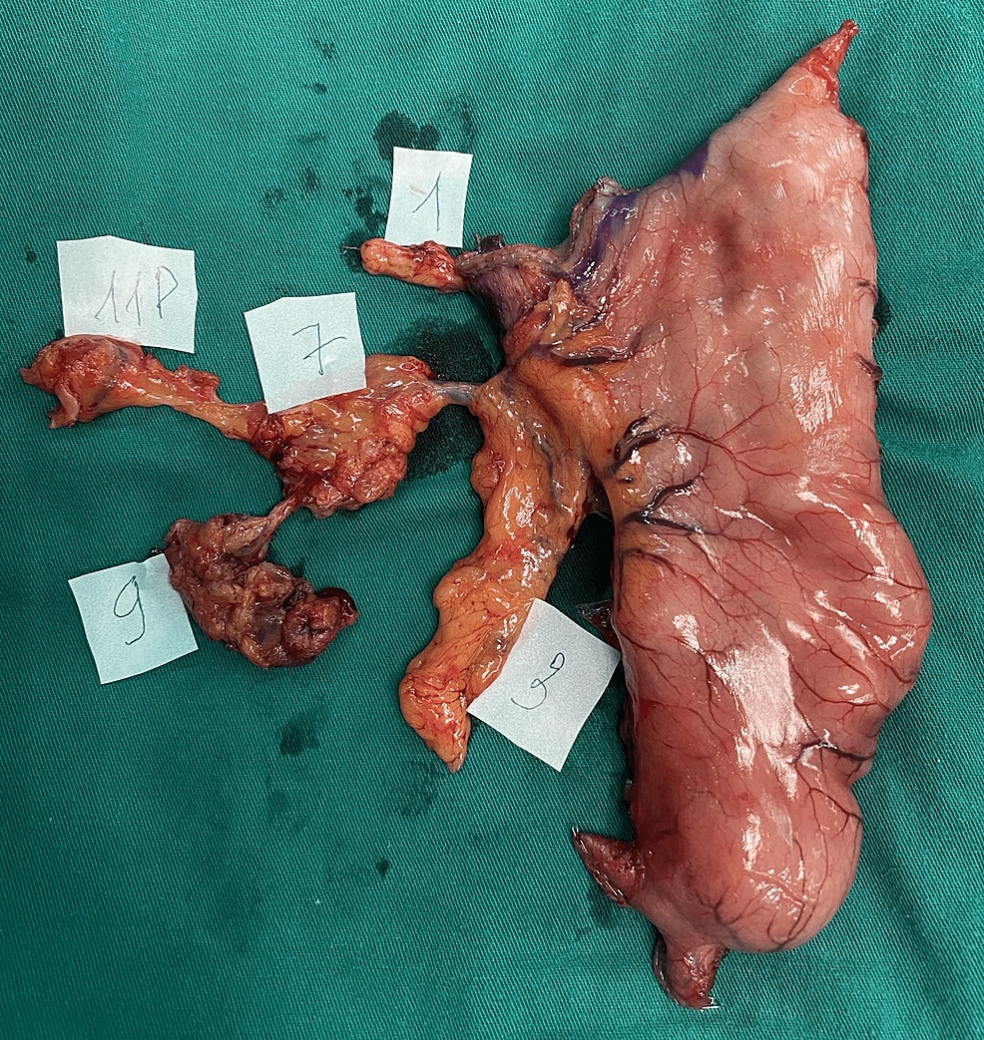
Lymph node station No. 1, No. 3, No. 7, No. 9, and No. 11p from the specimen

According to intraoperative landmarks, one surgeon would manage the specimen and separate the LNs along with the surrounding connective tissue into separate LN station blocks (Figures 2-4). These blocks would be put into each labeled pot, fixed with neutral formalin, and then sent to the pathologists. The pathologists then managed these blocks with specific chemicals to retrieve LNs and then investigate each of those LNs, along with the tumor. Pathology reports would be returned to surgeons about five days after surgery.

Statistical analysis

Demographic data included age, gender, tumor location (longitudinal and cross-sectional), and macroscopic classification. Histopathological data included differentiation, lymphatic invasion status, vascular invasion status, number of total LNs retrieved, number of LNs for each station, the total number of mLNs, and the number of mLNs for each station. The mLN ratio was calculated in each case by the ratio between the number of mLN and the number of total LNs retrieved. We compare the difference in the total LN retrieved, number of mLN, and rate of positive LN (N+) between the two groups TG and sTG.

Descriptive analyses included mean and standard deviation for continuous variables, the number of patients, and percentages for categorical variables. Differences between the two groups concerning preoperative, intraoperative, and postoperative characteristics were tested using the Mann-Whitney U test for continuous variables and Fisher's exact test for categorical variables.

All study procedures were approved by the local Ethics Committee at our university and registered at researchregistry.com with the unique identifying number: researchregistry8766.

This cross-sectional study was reported in line with the Strengthening the Reporting of Cohort Studies in Surgery (STROCSS) 2019 Guideline (see appendix) [7].

## Results

The mean age was 60.8 ± 11.0 and males predominated (60.2%). Histopathology characteristics of 98 patients are demonstrated in Table 1. Most of the patients had advanced GC (82.7% pT2-4) with 60.2% serosa-involved GC. About 65.3% of patients had metastatic LN (pN+). There was a broad spectrum of cancer differentiation, including 20.4% of patients with signet ring cell carcinoma. Macroscopic findings consisted mainly of ulcerative lesions (Borrmann type 2 and type 3). There were 30.6% of patients with infiltrated patterns. For the longitudinal location of the tumor, there were 14.3% of patients with the upper third involved GC, which required TG. Eighteen patients (18.4%) with the middle-lower third GC underwent sTG, and 66 patients (67.3%) underwent either sTG or TG, depending on the safe proximal margin. The lesser curvature was still the most prominent position of GC. There were 11 cases (11.2%) with GC in the greater curvature and 16 cases (16.3%) with GC involving the whole circumference (Table 1).

**Table 1:** Pathology characteristics

Characteristics		N = 98, n (%n)
pT	pT1	17 (17.3)
	pT2	18 (18.4)
	pT3	4 (4.1)
	pT4a	59 (60.2)
pN	pN0	34 (34.7)
	pN1	16 (16.3)
	pN2	15 (15.3)
	pN3a	25 (25.5)
	pN3b	8 (8.2)
Differentiation	Well	6 (6.1)
	Moderate	25 (25.5)
	Poor	45 (45.9)
	Signet ring cell	20 (20.4)
	Mucus	2 (2.0)
Macroscopic findings	Type 0	15 (15.3)
	Borrmann type 1	4 (4.1)
	Borrmann type 2	49 (50.0)
	Borrmann type 3	20 (20.4)
	Borrmann type 4	10 (10.2)
Lymphatic/vascular invasion	No invasion	35 (39.3)
	Lymphatic invasion	8 (9.0)
	Vascular invasion	31 (34.8)
	Both	15 (16.9)
	Not assessed	9 (9.2)
Tumor location (longitudinal)	Middle only (M)	66 (67.3)
	Middle-lower (ML)	18 (18.4)
	Middle-upper (MU)	14 (14.3)
Tumor location (cross-sectional)	Lesser curvature	51 (52.0)
	Greater curvature	11 (11.2)
	Anterior quarter	11 (11.2)
	Posterior quarter	9 (9.2)
	Whole circumference	16 (16.3)

The distribution of metastatic LN for each station according to tumor invasion (pT) in the middle-third GC is shown in Table 2. The rate of LNs metastasis increased in correlation with the depth of tumor invasion. The events of LN metastasis and skipped LN metastasis happened even in tumors contained in the submucosal layer (pT1). In the serosal-involving (pT4a), most of the patients had metastatic LNs (92.5%). The metastasis rates in each LN station were also increasing in the same trend as the depth of tumor invasion. For LN station No. 2, 4sa, 10, 11d (which are not mandatory) in sTG, the rate of mLN was 0% when the tumor did not invade the serosa layer (pT1-3), regardless of tumor longitudinal location.

**Table 2:** Metastatic rate of lymph node stations according to tumor invasion (pT) * The number of survey cases. ** Percentage of metastatic lymph nodes. *** Median and interquartile range (IQR).

Station	T1		T2		T3		T4	
	N^*^	%^**^	N^*^	%^**^	N^*^	%^**^	N^*^	%^**^
1	17	0	18	16.7	4	0	59	28.8
2	2	0	6	0	1	0	32	18.7
3	17	0	18	27.8	4	50	59	54.2
4sa	2	0	6	0	1	0	32	18.7
4sb	17	0	18	0	4	0	59	16.9
4d	17	0	18	11.1	4	50	59	49.2
5	17	0	18	5.6	4	25	59	21.3
6	17	0	18	11.1	4	25	59	37.9
7	17	0	18	22.2	4	25	59	44.1
8a	17	0	18	5.6	4	25	59	23.7
9	17	5.9	18	0	4	0	59	18.6
10	1	0	3	0	1	0	27	3.7
11p	17	0	18	5.6	0	25	59	33.9
11d	2	0	6	0	1	0	32	6.3
12a	17	0	18	0	4	0	59	6.8
%mLN	17	5.8	18	44.4	4	50	59	92.5
LN^***^		0 [0;0] ^***^		0 [0;1] ^***^		3 [0;13]^ ***^		6 [2;11]^ ***^

The distribution of mLN for each station according to the cross-sectional location of the middle-third GC is shown in Table 3. The rate of mLN for each station tended to be higher in adjacent stations of the tumor (No. 1-3-5-7 in lesser curvature, No. 4sb-4d-6 in greater curvature, No.1-3-4sb in anterior wall tumors, and No. 3-7-12a in posterior wall tumors). In GC involving the whole circumference, which was usually large and highly advanced, the rates of mLN were relatively high in most of the stations, except for the No. 10 station.

**Table 3:** Metastatic rate of lymph node stations according to cross-sectional location of tumor * The number of survey cases. ** Percentage of metastatic lymph nodes. *** Median and interquartile range (IQR).

Station	Less		Great		Ant.		Pos.		Whole circumference	
	N^*^	%^**^	N^*^	%^**^	N^*^	%^**^	N^*^	%^**^	N^*^	%^**^
1	51	23.5	11	9.1	11	27.4	11	11.1	16	18.7
2	19	15.8	2	0	5	0	2	0	13	23.1
3	51	29.4	11	18.2	11	36.4	11	66.7	16	75
4sa	19	21.1	2	0	5	0	2	0	13	15.4
4sb	51	7.8	11	9.1	11	18.2	11	11.1	16	12.5
4d	51	23.5	11	45.5	11	27.3	11	22.2	16	68.7
5	51	11.8	11	9.1	11	18.2	11	11.1	16	25
6	51	17.6	11	36.4	11	36.4	11	33.3	16	37.5
7	51	27.5	11	36.4	11	27.3	11	55.5	16	31.2
8a	51	19.6	11	9.1	11	9.1	11	22.2	16	12.5
9	51	13.7	11	9.1	11	18.2	11	0	16	12.5
10	15	0	11	0	4	25	2	0	13	0
11p	51	15.7	11	27.3	11	27.3	11	11.1	16	43.7
11d	19	5.3	11	0	5	0	2	0	13	15.4
12a	51	5.9	11	0	11	0	11	11.1	16	0

We also conducted a sensitivity analysis of the mLN rates in No. 2, 4sa, 4sb, 10, and 11d for cases in which the tumor did not invade the upper third. The rates of mLN in these stations were 0% for pT1-3 tumors but were remarkably high (>5%) in T4a tumors (Table 4).

**Table 4:** Metastatic rate of lymph node stations 2, 4sa, 4sb, 10, 11d for tumor that did not invade into upper third (M, LM, ML) * The number of survey cases. ** Percentage of metastatic lymph nodes. *** Median and interquartile range (IQR). mLN: Metastatic lymph nodes; M: Middle only; LM: Lower-middle; ML: Middle-lower.

^Station^	^T1^		^T2^		^T3^		^T4^	
	^N*^	^%**^	^N*^	^%**^	^N*^	^%*^	^N*^	^%**^
^2^	^1^	^0^	^4^	^0^	^1^	^0^	^21^	^9.5^
^4sa^	^1^	^0^	^4^	^0^	^1^	^0^	^21^	^9.5^
^4sb^	^16^	^0^	^16^	^0^	^4^	^0^	^48^	^18.7^
^10^	^1^	^0^	^3^	^0^	^1^	^0^	^20^	^5^
^11d^	^1^	^0^	^4^	^0^	^1^	^0^	^21^	^9.5^
^Overall percentage of mLN^	^16^	^5.8^	^16^	^43.7^	^4^	^50^	^48^	^91.7^
^Overall median mLN^		^0 [0;0] ***^		^0 [0;3] ***^		^3 [0;13] ***^		^7 [2;11] ***^

Cases that went through two different procedures (sTG and TG) had different histological results as shown in Table 5. Those cases that required TG tended to locate in the upper two-thirds of the stomach or the large middle-third GC with deep invasion. The total LN retrieved, number of mLN, and rate of positive LN (N+) were statistically higher in the TG group compared to the sTG group. However, the mean mLN ratios were similar in the two groups (0.16 ± 0.03 in the sTG group and 0.23 ± 0.03 in the TG group, p = 0.116).

**Table 5:** A comparison of histopathological results between two procedures mLN: Metastatic lymph nodes; LN: Lymph node; IQR: Interquartile range.

		Near-total gastrectomy (n = 57)	Total gastrectomy (n = 41)	p-value
pT	T1	15 (26.3)	2 (4.9)	0.007
	T2	12 (21.0)	6 (14.6)	
	T3	3 (5.3)	1 (2.4)	
	T4	27 (47.4)	32 (78.1)	
pN	N0	28 (49.1)	6 (14.6)	0.004
	N1	9 (15.8)	7 (17.1)	
	N2	5 (8.8)	10 (24.4)	
	N3a	12 (21.0)	13 (31.7)	
	N3b	3 (5.3)	5 (12.2)	
Longitudinal location	Middle only (M)	51 (89.5)	15 (36.6)	0.001
	Middle-lower (ML)	6 (10.5)	12 (29.3)	
	Middle-upper (MU)	0 (0)	14 (24.1)	
Total LN retrieved	Median	22	34	0.005
	IQR	16-28	22-41	
Metastatic LN	Median	1	6	<0.001
	IQR	0-7	1-10	
mLN ratio	Mean (± SD)	0.16 ± 0.03	0.23 ± 0.03	0.116

## Discussion

In this study, we demonstrated a stratified distribution of mLN in the middle-third GC in accordance with the macroscopic and microscopic characteristics of the tumor. This was the first study on the Vietnamese population that details such information, especially focusing on the middle-third GC. It had been proven that mLN distribution or mapping was one of the most essential factors that affect the range of gastrectomy and the range of LN dissection in curative surgery [8-11]. In the middle third involved GC when surgery could be either TG or sTG or pylorus-preserving gastrectomy (PPG), this kind of data became even more important in establishing a recommendation for surgeons.

In the early middle-third GC, LN metastases were not seen in LN stations No. 5, No. 10, No. 11p, No. 11d, and No. 12a, which enhanced the indication of the PPG for these cases [4,12-14]. Some studies had suggested that PPG could be considered in T2 GC based on the remarkably low rate of mLN in LN station No. 5 and No. 6 [12-14]. Our study, however, could not jump to the same conclusion as mLN was seen in the No. 5 station of the pT2 tumor. We suggested that the indication of PPG should be carefully evaluated in pT2 middle-third GC, in terms of LN metastases.

In this study, we did not record LN metastases in LN station No. 4sb (39 cases), No. 2, 4sa, 10, and 11d (nine cases) when the tumor did not invade the serosa layer (pT1-3) (Tables 3, 4), which was different from previous studies [3,15]. When confirmed by bigger data, we proposed that for T1-T3 GC, No. 4sb LN dissection along the root of left gastro-epiploic vessels would not be mandatory (both TG and sTG), and LN No. 2, No. 4sa, No. 10, and No. 11d could be excluded in gastrectomy, as in sTG. For T4a GC, the rate of mLN was observed in almost all regional LN stations (Table 3). Even when the tumor did not invade the upper-third part, the LN metastasis was still seen in the cardia and splenic hilum areas (No. 2, No. 4sa, No. 10, and No. 11d) (Table 4). These results were similar to those of other studies in Japan, Korea, and Italy [3,15,16]. Thus, indicating sTG for such cases should also be carefully considered, especially in terms of mLN in the "leftover" LN station No. 2, 4sa, 10, and 11d (Tables 3, 4).

The distribution of mLN according to the cross-sectional location of the tumor is shown in Table 3. The rate of mLN for each station tended to be higher in the surrounding stations of the tumor. Since gastrectomy and lymphadenectomy were mostly dependent on the longitudinal location of the tumor rather than the cross-sectional one, these data would not make great changes in the determination of the types of procedures. However, some special aspects of LN dissection were still investigated, such as indications for N0 dissection. Several studies had proposed risk factors for No. 10 LN metastasis, including tumor size (>5 cm), infiltrated pattern (Borrmann 4), histological type of poorly differentiation or signet ring cell carcinoma, pT, pN, No. 4sa/4sb involvement, and greater curvature invasion [17-21]. In our study, the rate of No. 10 LN metastasis was relatively low, and such a correlation could not be accomplished. From our experiences, we suggested that No. 10 LN could be reserved in the pT1-T3 tumor that did not invade greater curvature, and no macroscopic No. 10 LN was suspected.

From our surgical strategy, TG would be the treatment of choice in cases that invade the upper-third part or a large middle-third GC in which a negative proximal margin was not provided. Table 5 demonstrates statistical differences in the aforementioned pT status, pN status, and longitudinal location. This, along with the increasing number of LN stations being retrieved (No. 2, 4sa, 10, 11d), results in statistical differences in the number of total LN retrieved and the number of mLN. However, the mean mLN ratios were similar in the two groups (Table 5). Several studies suggested that mLN ratios might be a better prognostic factor than the number of LNs [8-11,22]; this could be an interesting research topic in the near future.

This study had several potential limitations that could be resolved in the next studies. First, this kind of study should cover a greater number of cases, especially for LN station No. 2, 4sa, 10, and 11d. Second, long-term follow-up and survival data from these cases should be collected to estimate the therapeutic index for each LN station in each macroscopic and microscopic characteristic. These data could help us establish a GC LN mapping study and revise recommendations about the range of gastrectomy and LN dissection that fit the Vietnamese population.

## Conclusions

In accordance with the macroscopic and microscopic characteristics, we observed a stratified distribution of mLN in the middle third of the GC. With these early results, sTG combined with standard lymphadenectomy was an acceptable treatment for T1-T3 middle-third GC in terms of mLN distribution. Total No. 4sb LN dissection can also be reserved in gastrectomy for T1-T3 GC.

## Appendices

**Table 6:** Checklist for the STROCSS guideline Source: Ref. [7]. *Ref. [23].

The STROCSS Guideline		
Item no.	Item description	Page No.
Title		
1	Title: The word cohort or cross-sectional or case-controlled is included. The area of focus is described (e.g., disease, exposure/intervention, outcome). Key elements of study design are stated (e.g., retrospective or prospective).	1
Abstract		
2a	Introduction: The following points are briefly described - background and scientific rationale for this study.	2
2b	Methods: The following areas are briefly described - study design (cohort, retro-/prospective, single/multi-centered), patient populations and/or groups, including control group, if applicable, interventions (type, operators, recipients, timeframes), and outcome measures.	2
2c	Results: The following areas are briefly described - summary data (with statistical relevance) with qualitative descriptions, where appropriate.	2
2d	Conclusion: The following areas are briefly described - key conclusions, implications to practice, direction of and need for future research.	2
Introduction		
3	Introduction: The following areas are described in full relevant background and scientific rationale - aims and objectives, research question, and hypotheses, where appropriate.	3
Methods		
4a	Registration and ethics: Research registry number is stated in accordance with the declaration of Helsinki*. All studies (including retrospective) should be registered before submission. *Every research study involving human subjects must be registered in a publicly accessible database before recruitment of the first subject (this can be obtained from ResearchRegistry.com or ClinicalTrials.gov or ISRCTN).	4-6
4b	Ethical approval: The following areas are described in full - necessity for ethical approval, ethical approval with relevant judgment reference from ethics committees; where ethics was unnecessary, reasons are provided.	6
4c	Protocol: The following areas are described comprehensively - protocol (a priori or otherwise) details, with access directions; if published, journal is mentioned in the reference provided.	4-5
4d	Patient involvement in research: Describe how, if at all, patients were involved in study design, e.g., were they involved in the study steering committee, did they provide input on outcome selection, etc.	4
5a	Study design: The following areas are described comprehensively - “Cohort” study and design (e.g., retro-/prospective, single/multi-centered).	4
5b	Setting: The following areas are described comprehensively - geographical location nature of institution (e.g., academic/community, public/private), dates (recruitment, exposure, follow-up, data collection).	4
5c	Cohort groups: The following areas are described in full - number of groups and division of intervention between groups.	4
5d	Subgroup analysis: The following areas are described comprehensively - planned subgroup analyses, methods used to examine subgroups and their interactions.	5
6a	Participants: The following areas are described comprehensively - eligibility criteria, recruitment sources, and length and methods of follow-up.	5
6b	Recruitment: The following areas are described comprehensively - methods of recruitment to each patient group and period of recruitment.	5
6c	Sample size: The following areas are described comprehensively - margin of error calculation, analysis to determine the study population, power calculations, where appropriate.	
Intervention and considerations		
7a	Pre-intervention considerations: The following areas are described comprehensively - patient optimization (pre-surgical measures) and pre-intervention treatment (hypothermia/-volemia/-tension; ICU care; bleeding problems; medications).	5
7b	Intervention: The following areas are described comprehensively - type of intervention and reasoning (e.g., pharmacological, surgical, physiotherapy, psychological), aim of intervention (preventative/therapeutic), concurrent treatments (antibiotics, analgesia, anti-emetics, NBM, VTE prophylaxis), and manufacturer and model details, where applicable.	5
7c	Intra-intervention considerations: The following areas are described comprehensively - administration of intervention (location, surgical details, anesthetic, positioning, equipment needed, preparation, devices, sutures, operative time). Pharmacological therapies include formulation, dosages, routes, and durations. Figures and other media are used to illustrate.	5
7d	Operator details: the following areas are described comprehensively - training needed, learning curve for technique, specialization and relevant training	
7e	Quality control: The following areas are described comprehensively - measures taken to reduce variation, measures taken to ensure quality and consistency in intervention delivery.	5
7f	Post-intervention considerations: The following areas are described comprehensively - postoperative instructions and care, follow-up measures, future surveillance requirements (e.g., imaging, blood tests).	5
8	Outcomes: The following areas are described comprehensively - primary outcomes, including validation, where applicable, definitions of outcomes, secondary outcomes, where appropriate, follow-up period for outcome assessment, divided by group.	5
9	Statistics: The following areas are described comprehensively - statistical tests, packages/software used, and interpretation of significance, confounders and their control, if known, analysis approach (e.g., intention to treat/per protocol), and sub-group analysis, if any.	5
Results		
10a	Participants: The following areas are described comprehensively - flow of participants (recruitment, non-participation, cross-over and withdrawal, with reasons) and population demographics (prognostic features, relevant socioeconomic features, and significant numerical differences).	6
10b	Participant comparison: The following areas are described comprehensively. Table comparing demographic included differences, with statistical relevance, and any group matching, with methods.	
10c	Intervention: The following areas are described comprehensively - changes to interventions, with rationale and diagram, if appropriate, learning required for interventions, degree of novelty for intervention.	
11a	Outcomes: The following areas are described comprehensively - clinician-assessed and patient-reported outcomes for each group, relevant photographs and imaging are desirable, confounders to outcomes, and which are adjusted.	6-7
11b	Tolerance: The following areas are described comprehensively - assessment of tolerance, lost to follow-up, with reasons (percentage and fraction), and cross-over with explanation.	6-7
11c	Complications: The following areas are described comprehensively - adverse events described and classified according to Clavien-Dindo classification*.	
	Mitigation for adverse events (blood loss, wound care, revision surgery should be specified).	
12	Key results: The following areas are described comprehensively - key results, including relevant raw data, and statistical analyses with significance.	6-7
Discussion		
13	Discussion: The following areas are described comprehensively - conclusions and rationale, reference to relevant literature Implications to clinical practice, comparison to current gold standard of care, and relevant hypothesis generation.	7-8
14	Strengths and limitations: The following areas are described comprehensively - strengths of the study, limitations and potential impact on results, and assessment of bias and management.	9
15	Implications and relevance: The following areas are described comprehensively - relevance of findings and potential implications to clinical practice are detailed, future research that is needed is described, with study designs detailed.	8
Conclusion		
16	Conclusions: Key conclusions are summarized. Key directions for future research are summarized.	9
Declarations		
17a	Conflicts of interest: Conflicts of interest, if any, are described.	9
17b	Funding: Sources of funding (e.g., grant details), if any, are clearly stated.	9
